# Behçet's Disease Presenting With Large Intracavitary Thrombi and Pulmonary Embolism

**DOI:** 10.7759/cureus.95364

**Published:** 2025-10-25

**Authors:** Srinath TS, Shilpa S Menon, Raniya Palliyedath, Thilagavathi N

**Affiliations:** 1 Interventional Cardiology, SIMS Hospitals, Chennai, IND; 2 Cardiology, SIMS Hospitals, Chennai, IND; 3 Rheumatology, SIMS Hospitals, Chennai, IND

**Keywords:** behcet’s disease, computed tomography, rare chronic inflammatory disorder, right ventricular thrombi, thrombolysis

## Abstract

Behçet's disease (BD) is a rare chronic multisystem inflammatory disorder characterized by recurrent mucocutaneous, ocular, musculoskeletal, and vascular manifestations. Although vascular involvement is common, cardiac manifestations are exceptionally rare and can be life-threatening. We report a unique case of a 24-year-old male who presented with haemoptysis and right-sided chest discomfort. Echocardiography demonstrated multiple right ventricular thrombi, and a CT pulmonary angiogram confirmed extensive thrombi with near-total occlusion of the left pulmonary artery. Despite hemodynamic stability, the significant thrombus burden and presence of right ventricular thrombus prompted thrombolysis with intravenous Alteplase, resulting in clinical improvement, followed by anticoagulation therapy. Further workup, including HLA-B51 testing and the presence of recurrent folliculitis, supported a diagnosis of Behcet’s disease. This report emphasizes the importance of early recognition and combined immunosuppressive and anticoagulant therapy to manage this rare but fatal presentation.

## Introduction

Behçet's disease (BD) is named after Hulusi Behçet, a Turkish dermatologist who initially described a triple symptom combination of aphthae, genital ulcers, and hypopyon uveitis in three individuals in 1937 [1]. The hallmark of the disease is recurrent oral aphthae, considered its essential defining feature, accompanied by other recurrent manifestations in order of decreasing frequency: genital ulcers, various skin lesions, arthritis, uveitis, and thrombophlebitis [2]. BD has a distinct geographic distribution along the historical trade route referred to as the “Silk Route,” which spans from Mediterranean nations to the Far East. This pattern suggests that its causative factors, including multiple genetic elements such as human leukocyte antigen (HLA)-B51, may have been disseminated along this route [3].

The prevalence of vascular involvement in BD is 7-43% globally and is usually associated with recurrent deep vein thrombosis of the lower limbs, vena cava thrombus, and pulmonary artery aneurysm [4]. This condition is uncommon after the age of 50 and rare in children. While BD affects both genders equally, the disease generally follows a more severe course in men and in younger patients [5]. Cardiac involvement in BD is rare and can be life-threatening. The presence of intracavitary thrombi and pulmonary embolism in an otherwise healthy, young male is unusual. The case we present here is particularly notable because the patient presented to the outpatient department (OPD) in a relatively stable condition. To date, only two comparable cases have been reported from India.

## Case presentation

A 24-year-old male presented to the OPD with a history of intermittent mild hemoptysis for one month. He also complained of right-sided chest discomfort that was pleuritic in nature. He had previously consulted a local physician who treated him with a course of antibiotics. He had been referred to a pulmonologist for persistent symptoms, and a CT chest performed elsewhere had revealed patchy subpleural consolidation. On examination in the OPD, the patient was hemodynamically stable with a pulse rate of 90/min and blood pressure of 110/70 mmHg. He was not tachypneic and had a normal oxygen saturation of 97% on room air. Jugular venous pressure was not elevated, and there was no pedal edema. Systemic examination was unremarkable. There was no loud P2 component of the second heart sound and no added sounds on lung auscultation.

**Figure 1:**
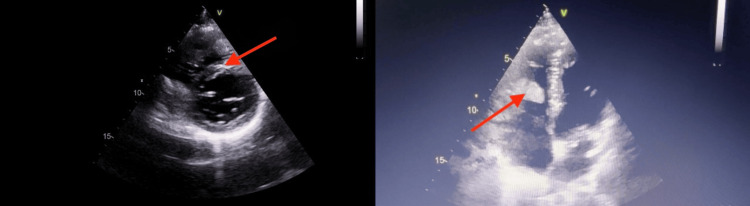
Echocardiographic images showing right ventricular thrombi in the parasternal short-axis and apical four-chamber views (arrows)

**Figure 2:**
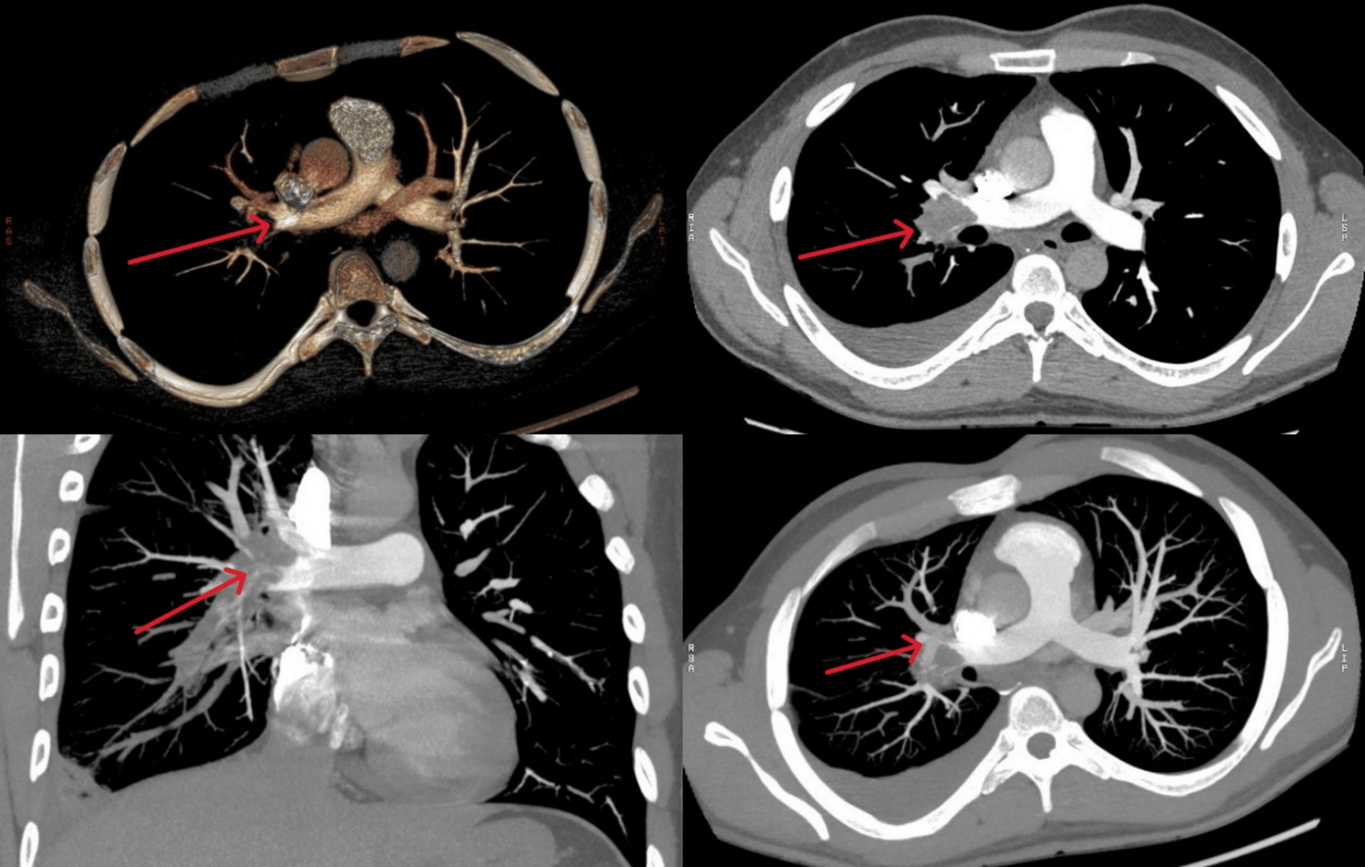
CT pulmonary angiogram The images show right atrial and ventricular thrombi, bilateral pulmonary artery thrombi with subsegmental emboli, right subpleural consolidation, and near-total cutoff of the left pulmonary artery (arrows) CT: computed tomography

Echocardiography performed at our center revealed multiple right ventricular (RV) thrombi, the largest measuring 21 × 39 mm, along with a small thrombus in the superior vena cava-right atrial (SVC-RA) junction and an additional thrombus within the right pulmonary artery (Figure 1). The RV function and estimated pulmonary artery pressure were normal. A CT pulmonary angiogram performed the same day showed RV and right atrial thrombi, bilateral pulmonary artery thrombus, subsegmental pulmonary embolism, right subpleural consolidation, and near-total cutoff of the left pulmonary artery (Figure 2).

Despite being hemodynamically stable, the heavy thrombus burden on CT, near-total cutoff of the left pulmonary artery, and the presence of RV thrombus led us to thrombolyze the patient with alteplase. He was thrombolyzed after samples were drawn for a procoagulant workup panel that included protein C, protein S, lupus anticoagulant, Factor V Leiden mutation, and antithrombin III levels. He tolerated thrombolysis well and was observed in the critical care unit for 24 hours afterward. He remained hemodynamically stable. Low molecular weight heparin, bronchodilators, and antitussives were administered.

The patient had recurrent folliculitis on his face, genitals, and thighs and had been on oral isotretinoin for two years. Isotretinoin was discontinued in consultation with a dermatologist. A positron emission tomography-CT (PET-CT) scan revealed no signs of malignancy. A vasculitis panel was sent in consultation with a rheumatologist, along with serum homocysteine levels, HLA-B51, and anti-beta-2 glycoprotein (IgG and IgM). Results revealed elevated serum homocysteine, HLA-B51 positivity, mildly positive lupus anticoagulant, and activated protein C resistance. He was started on warfarin along with folic acid, pyridoxine, and cyanocobalamin supplementation. A rheumatologist was consulted, and initiating azathioprine at the follow-up visit was planned. Additionally, an ophthalmologist was consulted to exclude the presence of asymptomatic uveitis. A skin pathergy test and thiopurine methyltransferase test were planned during outpatient review. The patient was discharged.

**Figure 3:**
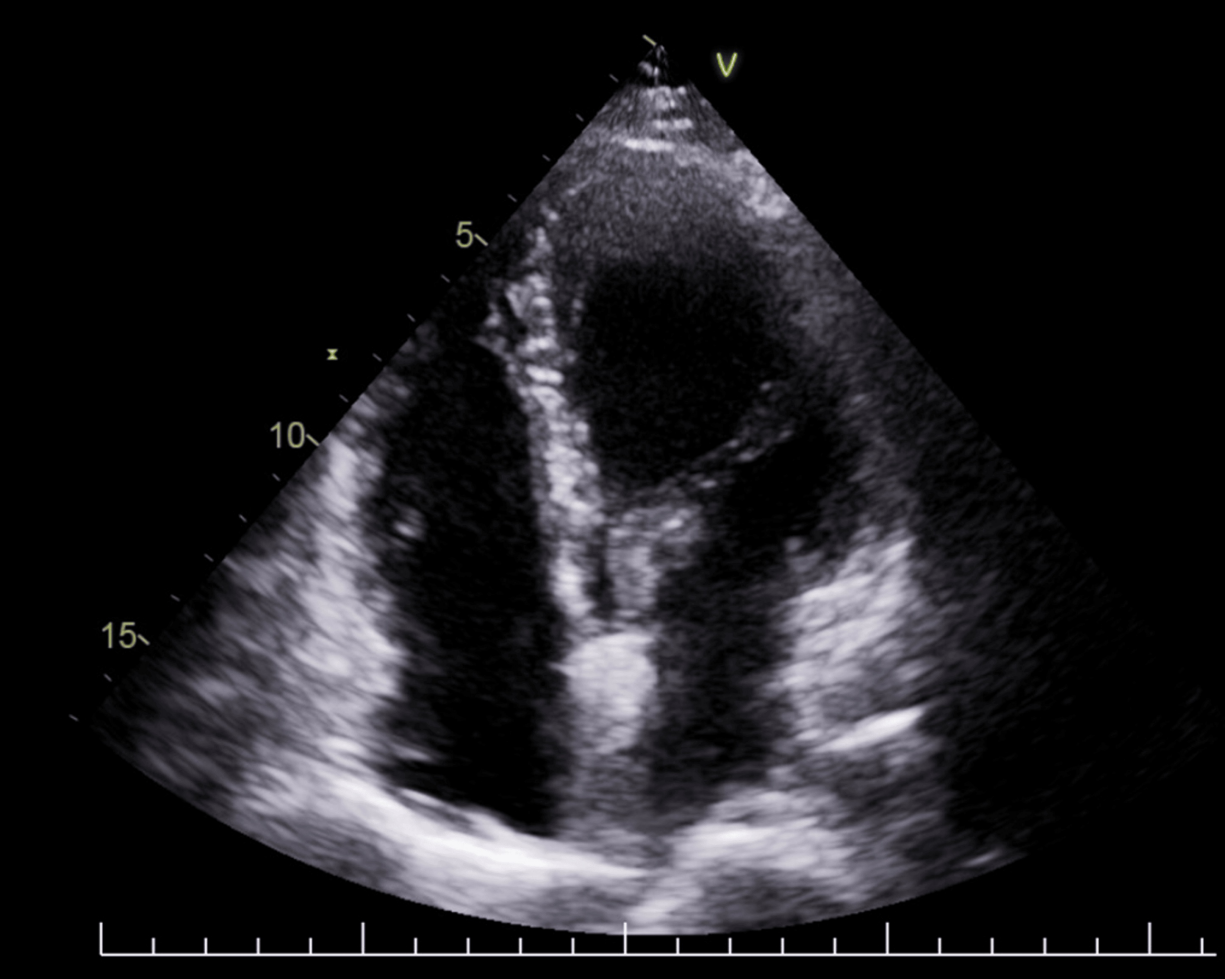
Repeat echocardiogram at 3 months post-discharge showing complete resolution of right atrial and right ventricular thrombi

The patient was subsequently started on oral prednisolone, azathioprine, and colchicine. Folic acid, pyridoxine, and cyanocobalamin supplementation were continued. Warfarin was stopped after three months following complete resolution of intracavitary thrombi (Figure 3).

## Discussion

The diagnosis of BD is based on clinical criteria. The International Study Group (ISG) criteria and the revised International Criteria for Behçet's Disease (ICBD) prioritize the presence of recurring oral ulcers and other signs, including genital ulcers, eye involvement, skin lesions, or a positive pathergy test [2]. Our patient was diagnosed with BD based on mucocutaneous lesions, the presence of thrombi in the right atrium and ventricle, pulmonary embolism, and HLA-B51 positivity. Intracardiac thrombosis is a serious complication with poor prognosis and often occurs in association with pulmonary artery aneurysm (42%), pulmonary thromboembolism (52%), and venous thrombosis (56%) [6]. Patients predominantly complain of fever, hemoptysis, dyspnea, and cough at the time of presentation. Young men in the third decade of life are most commonly affected, with the right heart being the most frequently involved site.

Treatment of BD depends on the severity, duration, and organ systems involved, often requiring individualized treatment regimens. Oral and genital ulcers can be treated with topical corticosteroids [7]. While erythema nodosum and genital ulcers can be prevented from recurring, colchicine remains the first-choice systemic treatment for mucocutaneous lesions [8]. In cases of intracardiac thrombosis, patients are treated with a combination of anticoagulants, immunosuppressants, and colchicine. Surgical resection has also been performed in cases of large thrombi not responding to medical therapy [9]. Remission of cardiac involvement has been associated with the use of immunosuppressants and colchicine.

This report highlights the importance of conducting comprehensive clinical assessments and maintaining vigilance when treating young patients with intracardiac thrombi. Identifying the primary underlying etiology and initiating appropriate treatment can prevent relapses and life-threatening complications.

## Conclusions

BD is a multisystem vasculitis with diverse clinical manifestations, and cardiac involvement remains one of its rarest and most life-threatening complications. This report highlights the unusual presentation of extensive intracardiac thrombi and pulmonary embolism in a hemodynamically stable young male, emphasizing that significant clot burden, even in the absence of hemodynamic instability, may warrant early intervention. Prompt recognition and a multidisciplinary approach involving cardiology, pulmonology, rheumatology, and dermatology were essential for accurate diagnosis and management. The combination of immunosuppressive therapy with short-term anticoagulation led to complete resolution of thrombi and clinical stability. Clinicians should maintain a high index of suspicion for BD in young patients presenting with unexplained or recurrent thrombotic events, especially when these occur at unusual sites or show resistance to standard anticoagulation. Early diagnosis and tailored immunomodulatory treatment can prevent recurrence and significantly improve outcomes.
